# Exploring the landscape of public attitudes towards gene-edited foods in Japan

**DOI:** 10.1270/jsbbs.23047

**Published:** 2024-02-22

**Authors:** Tomiko Yamaguchi, Kazune Ezaki, Kyoko Ito

**Affiliations:** 1 College of Liberal Arts, International Christian University, 3-10-2 Osawa, Mitaka, Tokyo 181-8585, Japan; 2 Department of Life Science, College of Science, Rikkyo University, 3-34-1 Nishi-ikebukuro, Toshima-ku, Tokyo 171-8501, Japan; 3 Faculty of Engineering, Kyoto Tachibana University, 34 Yamada-cho, Oyake, Yamashina-ku, Kyoto 607-8175, Japan; 4 Graduate School of Information Science and Technology, Osaka University, 1-5 Yamada-oka, Suita, Osaka 565-0871, Japan

**Keywords:** social acceptance, public attitudes, gene-edited foods, naturalness

## Abstract

The success or failure of food technologies in society depends to a large extent on the public interest, concerns, images, and expectations surrounding them. This paper delves into the landscape of public attitudes towards gene-edited foods in Japan, exploring the reasons behind the acceptance or rejection of these products. A literature review and preliminary findings from a survey conducted in Japan in 2022, aim to identify key issues crucial for evaluating societal acceptance of gene-edited foods. The study showed that the public view gene-edited foods as somewhat unnatural, but upon closer examination, significant variation in attitudes was observed among respondents. Some respondents expressed a favorable perception towards gene-edited foods, particularly those that benefit consumers, while others expressed concerns about its perceived artificiality. Moreover, a significant number of respondents displayed indifference or lack of clear perspective regarding gene-edited foods. These findings reflect the complex relationship between public attitudes, naturalness, and social acceptance of gene-edited foods. Furthermore, the study indicates the importance of paying close attention to those who refrain from expressing their viewpoints in the survey. This nuanced landscape warrants further exploration.

## Introduction

The success or failure of food technologies in society depends to a large extent on the public interest, concerns, images, and expectations surrounding them. The European experience with genetically modified organisms (GMOs) provides relevant insights as studies suggest that weak or non-existent public support leads to tensions and even a decline in trust in science (Frewer *et al.* 2004, Klintman 2002, Pew Research Center 2016). This is particularly true for technologies that have previously been socially controversial, as the opinions that the public previously formed will play a crucial role in shaping the future trajectories. Gene-edited foods are an example of such a case, as they have been associated with intense social controversies over GMOs in the past (Cummings and Peters 2022). Japan is unlikely to be an exception to these phenomena. Therefore, understanding and addressing public interests and concerns is essential for the successful integration of gene-edited foods into society. Gene editing is a powerful tool that can help improve food production and nutrition. However, it is necessary to ensure that the technologies are consistent with the norms and values of society before their integration into society.

Within Japan both scientific communities and public policy circles recognize that the implementation of gene editing technologies is highly dependent on public understanding, and support for gene-edited foods (Science Council of Japan 2014, 2017). Developers of food technologies also acknowledge that public reservations regarding their products can lead to delays in commercialization and diffusion (Japan Bioindustry Association 2018). Thus, the notion of “social acceptance” has gained prominence in Japanese public policy discussions, particularly with regards to advanced scientific and technological development. However, its usage frequently lacks precision, with an excessive focus on abstract notions and broad concerns, which makes it challenging to discern its practical implications. Various interpretations of the term social acceptance exist among stakeholders, including acceptance of the technology itself, its regulatory framework, specific projects, or products and services that use the technology. These differing interpretations may result in social conflicts and confusion. Additionally, the term “acceptance” is inadequate for explaining the complex underlying mechanisms required for its manifestation (Alexandre *et al.* 2018, Spök *et al.* 2022). It is crucial to recognize that social acceptance is a multifaceted phenomenon that involves various factors, such as legal, social, cultural, and economic considerations. Recognizing the complexity of this issue, this analysis focuses on the attitudes of the Japanese public towards gene-edited foods and the reasons for their acceptance or rejection of these products. Through this exploration, we aim to identify key issues that are essential in assessing societal acceptance of gene editing technology.

To better explain the social acceptance issues surrounding gene editing technology, we will provide a literature review and ground our discussion in the preliminary findings from a survey conducted in Japan in 2022. Analyzing specific technologies within their socio-cultural context will uncover crucial insights into key issues regarding social acceptance of gene-edited foods. First, we will provide a contextual background on gene-edited food and crops specific to Japan. Next, we will conduct a literature review on the aspects influencing social acceptance. We’ll begin by examining literature related to the public’s expectations and concerns regarding gene-edited foods and related technologies, the concept of naturalness concerning food and its associated concerns, as well as the influences of information and knowledge on attitudes. Finally, we will present preliminary findings obtained from our survey which investigated 13 different gene-edited foods and crops.

## Japan’s Gene Editing Landscape

Genetically modified (GM) crops have rapidly gained significance in global agriculture, with commercial cultivation spanning numerous countries since their inception. By 2019, GM crops covered approximately 190.4 million hectares, cultivated by 17 million farmers across 29 countries (ISAAA 2019). While numerous nations have adopted GM crops for enhanced productivity, Japan has taken a more precautionary approach, imposing stricter regulations on transgenic crops in comparison to countries like the United States, Canada, and Australia. As a result, the cultivation of GM crops in Japan has been slow to progress. Although the planting of GM crops is legally permissible, commercial cultivation has been limited. Interestingly, while domestic cultivation is limited, Japan is a major importer of GM crops. It imports nearly all of its corn and a significant portion of its soybeans, which are genetically modified (USDA FAS 2020).

Japan’s regulatory framework includes four ministries: the Ministry of Agriculture, Forestry and Fisheries (MAFF), the Ministry of Health, Labour and Welfare (MHLW), the Ministry of Environment (MOE), and the Ministry of Education, Culture, Sports, Science, and Technology (MEXT). The Food Safety Commission (FSC), an independent assessment body, oversees food and feed safety risk assessments for MHLW and MAFF. In 2003, Japan ratified the Cartagena Protocol on Biosafety, which led to the enactment of the ‘Cartagena Act.’ This act aims to regulate living modified organisms and conserve biodiversity (Ministry of Justice 2003). Beyond national regulations, some local governments have taken additional steps by implementing ordinances that limit the commercial cultivation of GM crops (Sassa 2021). Interestingly, despite approving a number of GM events second only to the US, Japan maintains a precautionary approach to GM plant commercialization (The Law Library of Congress 2014).

In 2019, in light of the Cartagena Act, Japan’s Ministry of Environment established policies to regulate gene-edited organisms (Notification No. 1902081, Ministry of Environment 2019). These policies clarified handling procedures for gene-edited products for biosafety. Simultaneously, procedures for handling of the safety of gene-edited feed and food have also been finalized (USDA FAS 2020). With particular reference to the biosafety policy, the Central Environment Council of Japan advised that only gene edited organisms with foreign genes or their fragments added will be regulated. As a result, some gene-edited crops, such as those derived from SDN1-type events, remain unregulated (Tsuda *et al.* 2019). In parallel development, the Consumer Affairs Agency decided not to require labels for gene-edited foods, on the grounds that these products are scientifically indistinguishable from conventionally bred products. This decision was seen as deregulation by some consumer advocacy groups and sparked public debate.

The intricate landscape of genetically modified crops in Japan highlights a distinct approach to the adoption and controlling gene-edited foods and crops. This blend of thorough examination, precise legal definitions, and evolving guidelines typifies Japan’s stance towards both genetically modified crops and gene editing technologies. As gene editing technologies have emerged, so as Japan’s approach to these innovations, highlighting the importance of comprehending this landscape in order to understand public perspectives.

## Aspects of Social Acceptance

### Expectations and concerns

Understanding the expectations and concerns of the public is a crucial first step in assessing the social acceptability of gene-edited foods. This would allow for a better understanding of the issues that need to be considered when attempting to assess social acceptability. Views on gene-edited foods vary, with some people optimistic and others skeptical. Those who see novel food technologies in a positive light believe that they have great potential to achieve goals such as significantly reducing environmental impacts compared to traditional approaches (Tuomisto and Teixeira de Mattos 2011). Others believe that the introduction of gene-edited foods offers opportunities to address global food poverty (Haque *et al.* 2018). In addition, some suggest that gene-edited foods may offer numerous benefits such as mitigating the effects of climate change, increasing crop productivity, enhancing nutritional profiles, and reducing reliance on pesticides (Abdallah *et al.* 2015, Georges and Ray 2017, Karavolias *et al.* 2021, Yadav *et al.* 2021).

However, these claimed benefits have been met with skepticism. Some critics continue to question the effects of gene-edited foods and crops (Canadian Biotechnology Action Network 2020, Helliwell *et al.* 2019). In the midst of conflicts surrounding GMOs, concerns have been raised about equitable access to technologies and the fair distribution of benefits. The use of GMOs has led to situations where the use of these technologies has primarily benefited large corporate farms and industries (Howard 2021). Along similar lines, concerns have been raised about the use of gene editing technologies or the potential use of cellular agriculture, which may advance the economic interests of specific groups, including the agricultural or food industries, often with little regard for the broader public interest (Courtier-Orgogozo *et al.* 2017, Howard 2022). Furthermore, unease about the ‘unnatural’ nature of GMOs is echoed in discussions of gene editing and synthetic biology (Thompson 2020). In particular, the concept of naturalness is important to many consumers and underlies their reservations about the use of technology in food production (Jorge *et al.* 2020, Nales and Fischer 2023), which may extend to gene-edited foods. In short, these expectations and concerns require careful consideration of how people perceive and evaluate the introduction of gene-edited foods into society. This will ensure that the decisions about their introduction are consistent with broader societal values and norms, taking into account all expectations and concerns.

### Naturalness

To gain a deeper understanding of the public’s expectations and concerns, it is crucial to examine how the concept of ‘naturalness’ affects consumer perceptions and, in turn, their acceptance of these technologies. This is particularly relevant as the concept of naturalness evokes almost exclusively positive emotions, while perceived unnaturalness is a key factor in resistance to emerging agricultural products (Hibino *et al.* 2023). This dichotomy between natural and unnatural has significant implications for the societal integration of gene editing technologies in agricultural and food production. This impact is further highlighted by research indicating that consumers often rely on the “natural-is-better” heuristic when evaluating novel food technologies. If a technology is perceived as unnatural, it tends to be less accepted (Siegrist and Hartmann 2020). Moreover, natural foods are inherently associated with notions of being healthier, tastier, and more environmentally friendly (Tenbült *et al.* 2005). Building on this, Sodano *et al.* (2016) extend the point by finding that perceptions of naturalness significantly influence an individual’s risk perception. Taken together, these findings highlight the potential importance of naturalness in shaping attitudes toward gene-edited foods and crops. Observations from Japan reveal that consumers tend to view gene-edited foods as less natural and more similar to genetically modified foods, especially when compared to crops developed through other breeding methods such as chemical mutagenesis or irradiation (Otsuka 2021). Other studies support this finding, suggesting that gene-edited foods are often associated with genetic modification, which may lead to a segment of the population rejecting their use (Ishii 2017). This underscores the complexity of considering the intricate role that the sense of ‘naturalness’ plays in forming opinions about food. It also highlights the need for further research in this area.

On the other hand, studies indicate that when comparing genome editing with transgenic genetic modification, respondents showed greater acceptance of gene-edited foods compared to genetically modified foods (Bearth *et al.* 2022, Beghin and Gustafson 2021). This perception holds true not only in the United States but also in the UK and several European countries. Research indicates that gene-edited foods are preferred over genetically modified organisms (Food Standards Agency 2021, Norwegian Biotechnology Advisory Board 2020). As a result, it is commonly believed that the public generally holds more positive attitudes toward genome editing as opposed to genetic modification. However, it is noteworthy that the difference in attitudes towards gene editing and genetic modification is smaller compared to the difference in attitudes toward conventional breeding and genetic modification (Kato-Nitta *et al.* 2019). Similarly, in comparing consumers’ wiliness-to-consume and pay for CRISPR-produced food with conventional and genetically modified foods, Shew *et al.* (2018) found that respondents generally valued CRISPR and GM foods similarly, but substantially less than conventional food.

Building on these perceptions of gene editing’s precision and preference, additional insights can be obtained by exploring people’s varying feelings toward transgenic and cisgenic gene modifications. This highlights the significant influence of naturalness perception in shaping social acceptance. Various attitudes arise regarding transgenic and cisgenic gene modifications. Empirical studies show that transgenics, or the crossing of species boundaries, is generally regarded as less natural compared to cisgenics, which involve the integration of genes from closely related or identical species (Dayé *et al.* 2023, Kronberger *et al.* 2013). This distinction is supported by European research, which suggests that respondents exhibit greater resistance toward transgenic modifications in contrast to cisgenic alterations (Delwaide *et al.* 2015). The idea that products resulting from cross-species processes are unnatural creates a sense of unease and contributes to public concerns. Expanding on this line of thought, De Marchi *et al.* (2021) draw similar conclusions and introduce an interesting aspect: highlighting the health and environmental benefits, especially the latter, tends to generate a more positive response toward cisgenic foods. However, another study has revealed that some cisgenic products are also categorized as genetically modified foods, resulting in demands from consumers for mandatory labeling (Kronberger *et al.* 2013).

Closely tied is the influence of the modification’s target species on the perception of naturalness. Research highlights that concerns are heightened when gene editing is applied to animals as opposed to vegetables, with the technology being perceived as riskier for animals (Bearth *et al.* 2022, Kato-Nitta *et al.* 2021). This pattern is mirrored in the study by Lusk *et al.* (2015), which unveils that consumers display greater resistance to consuming GM beef in comparison to GM corn or apples. These findings are consistent with Macnaghten’s (2004) empirical study on public attitudes towards genetic modification of animals, which found that one of the most common objections to the use of genetic modification is that it is wrong because it is unnatural. Further exploring this topic, Busch *et al.* (2021) conducted a study analyzing citizen perspectives on genome editing in five countries—Canada, the United States, Austria, Germany, and Italy. They found that disease resistance in humans was considered the most acceptable application of genome editing by the majority of research participants across all countries. Additionally, disease resistance in plants was the subsequent preference followed by disease resistance in animals. In contrast to these findings, a research consortium in Norway, composed of a university, government entities, and industries, conducted a study and found that respondents do not distinguish between animals and plants in their attitudes if the breeding purpose is the same (Norwegian Biotechnology Advisory Board 2020).

Furthermore, there are other related issues raised in the literature. For example, consumers might even harbor reservations about the integration of advanced technologies in packaging, potentially influencing their perception of a product’s naturalness (Greehy *et al.* 2011). Consumer preferences often lean towards packaging that solely features the food product, as the inclusion of additional elements could raise doubts about the product’s natural attributes. This sentiment extends to food additives as well. While these additives enhance the shelf life of packaged items, they tend to diminish the perceived naturalness of the products. van Wezemael *et al.* (2011) delve into how additives released by active packaging materials might be perceived in a manner akin to general food additives. These perceptions of additives, along with their connection to unnaturalness, can also extend to gene-edited crops, potentially influencing consumer acceptance and willingness to purchase such products. To sum up, the concept of perceived naturalness is a crucial aspect that significantly influences consumer attitudes toward a variety of food technologies, including gene-edited foods. This understanding becomes crucial in the effort to enhance their societal acceptance.

## Information/Knowledge and Attitudes

The impact of information and knowledge is a crucial factor in shaping public opinions regarding novel food technologies. Studies have shown that increasing people’s awareness of gene editing technologies significantly contributes to stimulating consumer acceptance (Shigi and Seo 2023). This is further corroborated by a recent survey conducted by a Consumer Group Liaison Meeting in Japan, which indicates that consumers themselves perceive a lack of information as contributing to a negative image of gene-edited foods (Consumers Japan 2022). Economic studies support these findings, noting that providing information about gene-edited foods enhances consumers’ willingness to pay for these products (Farid *et al.* 2020).

Extrapolating from the preceding conversation about GM foods, providing details on how genetically modified crops can solve societal issues is linked to positive perceptions and attitudes. This is particularly evident when the ecological benefits of genetically modified crops are highlighted (Saito *et al.* 2017). Supporting this, a 2021 survey conducted by the Council for Biotechnological Information Japan reveals that more than 50% of their respondents reported a positive change in their views towards GM foods after learning about how those technologies may aid in addressing socioeconomic and environmental concerns (CBIJ 2021).

However, certain types of information may actually lead to increased concern. For instance, the same 2021 CBIJ survey suggests that safety-related information plays a vital role in fostering a favorable perception, whereas technical information tends to generate a unfavorable views. This idea is echoed by studies indicating that increased exposure to scientific information does not necessarily alleviate negative attitudes (Irwin *et al.* 2013). For example, surveys conducted in Italy in 2000 and 2001 revealed that exposure to information does not always result in greater trust in biotechnologies, and that greater exposure to science in media does not guarantee an improved level of understanding (Bucchi and Neresini 2002). Valuable insights into the relationship between public attitudes toward GM foods and information can be gained from studies conducted in Japan in 2009. While more information generally increases social acceptance, an intriguing pattern emerges where acceptance initially rises, levels off, and subsequently declines (Sassa 2011, Tanaka 2009).

Examining the relationship between GMO-related knowledge and attitudes towards GMOs, Wunderlich and Gatto (2015) emphasize the importance of distinguishing between self-reported familiarity with GMOs and scientific understanding, the latter entails a deeper understanding of scientific principles. Their study revealed that familiarity with GMOs correlates with a preference towards non-GMOs and a higher willingness to pay for these alternatives. Conversely, higher scientific understanding is associated with fewer negative opinions of GM products, higher acceptance ratings, and less differentiation between various types of genetic modification. In short, studies indicate considerable disagreement on the issue, highlighting the importance of examining informational content.

While information and knowledge play a significant role in shaping attitudes towards gene-edited foods, the representation of science in mass and social media also has a crucial influence. According to a 2017 public opinion survey on science and technology by the Cabinet Office, a significant number of people obtain information on science and technology from television (83.2%), followed by newspapers (40.5%) and the internet (37.2%) (Cabinet Office, Government of Japan 2017). This highlights the importance of media coverage in connecting scientific developments with the public. In this context, the portrayal of gene-editing technologies through television, newspapers and the Internet is particularly influential. The media serves as a bridge between complex scientific concepts and the general public, translating these ideas into more digestible information. A recent study by Dawson *et al.* (2022) offers valuable insights into this issue. They found that favorable media coverage can significantly foster public support for the use of CRISPR. Interestingly, their results also revealed that exposure to science fiction television programming does not necessarily influence opinions regarding the application of gene editing. Social media also plays a significant role in shaping public opinion of gene-edited foods. Analyzing discussions surrounding gene editing technologies in agricultural contexts on both social media as well as news media in the United States, Hill *et al.* (2022) observed that tweets considerably influence public sentiment. Similarly, Tabei *et al.* (2020) examined Twitter conversations in Japan concerning genome-edited foods and highlighted the use of social media as a tool for monitoring public interests and opinions in real-time. Likewise, Wirz *et al.* (2021) conducted a study on Twitter discussions about genetically modified organisms (GMOs) in the United States and found that the topics of tweets can predict their sentiment, whether it is positive, negative, or neutral. If most tweets discuss potential health risks associated with gene editing technologies, then the sentiment is likely negative due to concerns and fears. Alternatively, if tweets focus on the potential benefits of gene editing technologies for agriculture, like increased crop yields or disease resistance, the sentiment might be more positive due to optimism and support generated.

Overall, these studies collectively highlight the complex relationship between information, knowledge, and individual attitudes towards gene-edited foods. By addressing these complex facets, we can develop a more comprehensive understanding of the social acceptability of gene-edited foods.

## Methods

In order to gain deeper insights into attitudes towards gene-edited products in Japan, this section presents some preliminary findings from a study where we conducted a survey in 2022. First, I will elaborate on the methodologies used. The survey aimed to assess participants’ perceptions regarding 13 distinct types of gene-edited crops and foods. A total of 1,111 individuals were surveyed using an online panel. We have taken account three factors when sampling: the regional population, the demographic distribution (with a criterion of age 18 years and over), and the gender distribution. These factors were chosen to reflect the distribution of the 2020 national census, ensuring that the data collected is representative. The survey was conducted using a structured questionnaire, with questions designed to elicit information on the participants’ attitudes related to gene-edited foods. The survey also included four open-ended questions to gain deeper insights into the reasons why a particular response category was chosen. Thirteen products were selected based on recommendations from scientists who are currently either involved in or considering involvement in the research and development of gene-edited foods either in universities or in industry. This methodological step was incorporated to identify products that are perceived by the Japanese scientific community as having a high potential for positive impact upon society. All responses were kept confidential. The data from the structured questions was analyzed to determine the frequency distribution, means, and standard deviation. The textual data collected from the open-ended questionnaires were coded via MAXQDA utilizing an after-coding method to identify new and unexpected themes emerging from the data. Any phrases which is open to two different interpretations were double-coded. The data was cross-checked for consistency by two researchers, one with a social science background and the other with a natural science background. This approach enables a multifaceted understanding of the data, improving interpretation and the reliability of the results. We did not evaluate inter-coder consistency numerically. Instead, in cases of disagreement, we proceeded with coding after discussions and reaching consensus. We categorized the preliminary codes into broader groups, enabling systematic data arrangement to contrast various reasoning behind people’s attitudes towards gene-edited foods.

## Results and Discussion

**Fig. 1.**
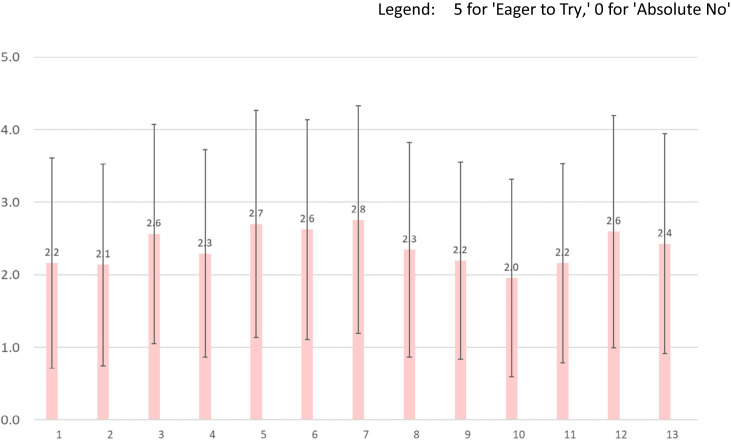
Average ‘Want to Try’ ratings by Product. This figure shows an average and standard deviation of ‘Want to Try’ ratings by Product. The data shows the percentage of respondents who rated their willingness to try gene-edited foods on a scale from 0 (Absolutely no) to 5 (Eager to try). The specific question posed was: ‘Regarding foods made using genome editing techniques, would you like to try eating them? Please choose one option for each of the following.’ Remarks: 1 = Red seabream with increased meat yield, 2 = High yield rice, 3 = Potatoes effective against dementia, 4 = Allergen free buckwheat, 5 = Tomatoes rich in carotene and lycopene, 6 = Wheat rich in fiber, 7 = Good-tasting rice, 8 = Pest resistent *Buri*, 9 = High-temperature tolerant rice, 10 = Docile tuna, 11 = Wheat tolerant to moisture, 12 = High-sugar melons, 13 = Caffeine-free teas; The graphs’ error bars represent the standard deviation. The product names listed above are literal English translations of the Japanese language names in the survey, not authentic scientific names.

In this section, we present the results of our study that aimed to evaluate the social acceptance of gene-edited food in Japan. We asked people their ratings of 13 products whether people want to try gene-edited foods. While our survey did inquire about respondents’ perspectives on the naturalness and commercialization of these products, we do not present those findings in this paper. We chose to focus on their responses to the ‘Want to Try’ question to highlight the most intuitive aspect of social acceptance. Fig. 1 shows an average and standard deviation of ‘Want to Try’ ratings by product. The data shows the percentage of respondents who rated their willingness to try gene-edited foods on a scale from 0 (Absolutely no) to 5 (Eager to try). In general, people hold a somewhat negative attitude toward trying new things, with the exception of five products. Interestingly, all of these exceptions are products that provide clear benefits to consumers not to producers. The highest average was good-tasting rice (2.8), followed by tomatoes rich in carotene or lycopene (2.7), potatoes effective against dementia (2.6), and high-sugar melons (2.6). We found the lower rating for high-yield rice (2.1), and docile tuna (2.0). Standard deviations of the 13 products showed that the responses were relatively dispersed for tomatoes rich in carotene and lycopene, good-tasting rice, and high-sugar melons, while the remaining items had lower dispersion.

These results suggest a nuanced picture of social acceptance of gene-edited foods in Japan. While there is a general reluctance to embrace gene-edited foods, there is also a clear openness towards products that offer tangible benefits to the consumer. This is evident in the higher ratings for products like good-tasting rice and high-sugar melons, which directly enhance the consumer’s experience. Interestingly, products that offer benefits primarily to producers, such as high-yield rice and pest resistant *Buri*, received lower ratings. Similarly, crops that can aid in mitigating the effects of climate change have received lower ratings as well. Additionally, the ratings for gene-edited fish were low. Based on these results, one can point out that consumers are more receptive to genome-edited foods that benefit them.

However, it’s important to note that even the highest-rated products did not receive overwhelmingly positive ratings. This indicates that while there is some level of acceptance at present, there is still considerable room for increasing public acceptance.

Shifting our focus to the question of why people are willing or not willing to try gene-edited foods, an open-ended question was included in a survey, and the results are outlined in Table 1. This table lists various reasons given by respondents, in addition to the total number and percentage of coded segments for each reason. The table is sorted by the percentage of coded segments in descending order so as to identify the most commonly cited reasons.

The table presents various reasons that either motivate or discourage people from trying these products. As demonstrated by the table, these reasons form the basis of the public’s expectations or concerns, which are crucial aspects of social acceptance as stated previously.

Analyzing the survey data on reasons for consuming or not consuming gene-edited foods, we found some interesting patterns. “For some reason or another” was the reason most frequently cited, accounting for 21.16% of the all the coded segments. “Indifferent” was the second most reason, with for 11.14% of the total. In addition, a similar theme of “not sure” emerged, representing 5.65% of the coded responses. Further examination of the views held by individuals in these codes reveals a range of perspectives. Some individuals express that they have “no particular reason” for their stance, while others indicate that their understanding of the issue is “underdeveloped” and therefore “not sure.” Some individuals simply rely on a “gut feeling” without providing a concrete rationale. A subset of respondents expresses “no interest in further exploring gene-edited foods.” Others find themselves unable to “actively identify a strong reason to consume” these foods. They believe that these crops lack benefits and do not offer any advantages. These comments suggest that individuals may be interested in, or hold reservations about, gene-edited foods, but they may have difficulty articulating their concerns. Alternatively, they may have a complex set of reasons that are difficult to summarize, or they may simply lack interest in these products. Consequently, their chosen words do not explicitly state their thoughts. Since these three items together occupy more than a third of all coded segments (37.95%), additional research is needed to identify people’s perspectives on this subject. It is noteworthy that a significant proportion of respondents often gravitated towards answers indicating indifference or neutrality. These findings align with the results of a survey conducted on synthetic biology and gene editing in Japan (Hibino *et al.* 2019). Their study also found a substantial portion of the population holding intermediate attitudes toward gene editing technology, further indicating a general trend of neutrality in responses. Collectively, these varied responses offer insights into public sentiment toward gene-edited crops. In considering the implementation of risk communication and science communication programs, it becomes essential to recognize this distinct tendency within the Japanese context.

The third most frequently reported reason was “unnaturalness or artificialness,” which accounted for 8.11% of total coded segments. This theme, which emerged with mostly negative connotations, indicates a prevalent concern among respondents. This perception of artificiality has led to a sense of unease among consumers, exacerbating their apprehensions about these foods. Several respondents expressed a clear preference for eating vegetables and fish in their natural state. This preference underscores the resistance to foods that are perceived as altered or manipulated in some way. These results demonstrate the obstacles in achieving social acceptance for gene-edited foods, as they are inevitably associated with an idea of unnaturalness and artificiality.

In addition to this, some respondents mentioned a strong desire to maintain their usual eating habits, reflected in their commitment to what they consider ‘normal’ food, underscoring the importance of familiarity in food consumption. Siegrist and Hartmann (2020) found that people tend to be conservative in their food choices, suggesting that the fundamental characteristics of food technology may conflict with this conservative thinking and impact social acceptance. Their research supports our findings, further emphasizing that the introduction of gene-edited foods appears to challenge their established dietary routines and preferences.

On the other hand, a significant number of optimistic comments were also made which are included in the codes “looks good for health” (7.0%) and “tasty” (6.6%). Many respondents expressed an interest in foods with higher nutritional value and indicated a willingness to try them. Specific comments revealed expectations for the listed foods to be functional, with specific health effects. For example, some respondents expressed a desire to try foods that were beneficial for dementia and for improving the bowel environment with enriched fiber. There was also interest in gene-edited foods that are caffeine free or that help to reduce allergic reactions. The word “tasty” was also frequently mentioned. Respondents commented on various benefits of the product, including its perceived superior taste compared to the regular variety. Some respondents expressed interest in trying the product if it was both safe and tasty, indicating that taste combined with safety are key factors influencing their willingness to try gene-edited foods.

While not as prevalent as other codes, it’s interesting to note that personal preferences for certain fruits and vegetables may influence social acceptance of gene-edited foods. For example, some respondents expressed a dislike for certain foods, with comments such as “I don’t like fish,” “I don’t want to eat melon,” or “I don’t like tomatoes.” Essentially, they were saying they didn’t want to eat these foods, whether they were gene-edited or not. In contrast, the survey also revealed 11 comments regarding foods that respondents already liked, indicating that some respondents were willing to try certain gene-edited versions of the foods they liked. Therefore, some people indicated their acceptance of certain gene-edited foods listed in the survey. Combined, these comments about personal food preferences accounted for 56 responses, or 4.46% of the total coded segments. This suggests that individual initial likes and dislikes may play a significant role in the acceptance of gene-edited foods.

Separately, a few respondents (1.03% of the total coded segments) in this study compared the application of genome editing technology in crop breeding and animal breeding. In particular, they expressed resistance to the use of this technology in animal breeding, including fish. Although research specifically comparing crop breeding and aquaculture is limited, our findings align with several previous studies comparing genetic modification in plants with animal breeding, primarily mammalian breeding (Hallman *et al.* 2004). Reservations about the gene modification in animals stem from a variety of reasons. A recent study by the Nuffield Council on Bioethics (2021) reveals that people’s concerns about gene alterations in animals vary depending on their perspectives. When respondents view themselves as potential consumers, their primary concerns center on product safety and the availability of relevant information to make informed purchase decisions. However, when they consider the issue from a citizen’s perspective, their concerns broaden to encompass the impact on the food system, farmed animals, social justice, and the environment. These perspectives form an integral part of the ethical considerations that guide people’s attitudes towards gene alterations in animals. On similar note, discussions, regarding animal welfare, disruption of the natural order, and potential unforeseen consequences could be the reasons for reservations for the gene modification in animals (de Graeff *et al.* 2019, Ishii 2017). These findings suggest that there are both frequently talked about worries as well as deep-seated concerns about gene modification of animals. This underlines the need to address these issues when considering the social acceptance of genome-edited foods.

Finally, it is noteworthy that the nomenclature of genome editing products emerged as a topic of comment among respondents. Some individuals found it challenging to understand the terms, indicating a potential gap in understanding or familiarity with the subject. Others expressed disapproval of the chosen names, suggesting a disconnect between the language used by the scientific community and the public’s understanding. These results highlight the choice of terminology can also influence public perception and acceptance. This also indicates the importance of clear and accessible language when introducing new technologies. The issue is not about the technology itself, but also how it is presented and communicated to the public. Efforts should be made to use language that is both scientifically accurate and easily understandable to non-specialists. This insight could be invaluable in shaping future strategies for promoting and implementing genome editing technologies.

## Conclusions

This paper explored public attitudes towards gene-edited foods, drawing on previous research and preliminary results from a 2022 survey conducted in Japan. The study reveals a multifaceted perspective shaped by factors such as crop breeding techniques, targeted organisms, and the interplay between information and public acceptance. The data indicate that gene-edited foods are generally perceived as somewhat unnatural. However, upon closer examination, significant variation in attitudes was observed among respondents, with some holding a positive outlook. These respondents expressed an interest in trying foods that are beneficial to their health and taste good. However, respondents also expressed apprehensions towards gene-edited foods due to perceived artificiality. These concerns stem from a heuristic that natural foods are superior, as well as conservative thinking when it comes to food choices. A substantial number of respondents in Japan were indifferent or did not have a clear opinion about gene-edited foods. These preliminary findings indicate the importance of paying close attention to these aspects associated with food technologies as we work towards responsible integration of innovative food technologies into our society.

## Author Contribution Statement

TY drafted the entire manuscript. KE conducted the data analysis, and KI contributed to the research design. All authors read and accepted the final version.

## Figures and Tables

**Table 1.** Reasons Why People are Wiliness to Consume or Avoid

Reasons	Coded segments of all documents (n)	Coded segments of all documents (%)
For some reason or another	266	21.16
Indifferent	140	11.14
Unnatural/artificial	102	8.11
Looks good for health	88	7.00
Tasty	83	6.60
Scary/anxious	74	5.89
Not sure	71	5.65
Seems bad for you	62	4.93
Want to try/Don’t want to try	56	4.46
Doubtful about safety or environmental impact	48	3.82
Dislike the food in the first place	45	3.58
Doesn’t seem to be tasty	42	3.34
Because it is genetically modified	32	2.55
Advantages	19	1.51
Allergy	17	1.35
Not particularly necessary	17	1.35
Food production	13	1.03
Crops vs. animals	13	1.03
Others	12	0.95
Favorite food to begin with	11	0.88
Disgusting	11	0.88
Curiosity	10	0.80
Depends on price	9	0.72
Trust	5	0.40
If it doesn’t affect my health	5	0.40
Naming	4	0.32
Made in Japan	2	0.16
Total	1257	100.00
